# Systematic Study of Some Epiphytic Algae (Non-diatoms) on the Submerged Parts of Water Hyacinth [*Eichhornia crassipes* (Mart.) Solms-Loubach] Found in Laguna de Bay, Philippines

**DOI:** 10.21315/tlsr2019.30.1.1

**Published:** 2019-01-31

**Authors:** Eldrin DLR. Arguelles

**Affiliations:** Philippine National Collection of Microorganisms, National Institute of Molecular Biology and Biotechnology (BIOTECH), University of the Philippine Los Baños, College, Laguna, Philippines, 4031

**Keywords:** Microalgae, Epiphytic Algae, Macrophyte, Systematics, Laguna de Bay

## Abstract

Taxonomic study on the composition of epiphytic algae living on submerged leaf and root tissues of macrophyte *Eichhornia crassipes* (Mart.) Solms-Loubach, found at Laguna de Bay, Philippines was conducted. In total, 21 algal taxa were identified: seven Cyanophyceae, six Euglenophyceae, five Chlorophyceae, two Trebouxiophyceae and one Klebsormidiophyceae. Of these taxa, the occurrence of two rare cyanobacteria, *Pseudanabaena minima* (G.S. An) Anagnostidis and *Synechococcus nidulans* (Pringsheim) Komárek are reported for the first time in the Philippines. Two species are also reported here for the first time in the Philippines based on current taxonomic nomenclature and these are *Pseudopediastrum boryanum* (Turpin) E. Hegewald, *Phormidium granulatum* (Gardner) Anagnostidis which were based on the former names of *Pediastrum boryanum* (Turpin) Meneghini and *Oscillatoria granulata* Gardner, respectively. These taxonomic records are considered important basal information in enriching the knowledge about the diversity and habitat distribution of cyanobacteria and microalgae on macrophytes found in freshwater habitats in the Philippines.

## INTRODUCTION

Epiphytic algae are group of algae found attached and living on submerged aquatic vegetation, which includes freshwater angiosperms and macroalgae (Dunn *et al*. 2008). These organisms are considered as primary source of food for small fish and several invertebrates in the littoral zone. These autotrophs are often over looked due to the tedious process of separation of epiphytons from the host plant; however, recent work showed their importance as primary producers that uptake essential nutrients and fix carbon from the water column, making these important nutrients accessible to other organisms such as small invertebrates and fish in the littoral zone (Ahmed 2010). Also, epiphytic algae serve as good indicators of water quality and environmental conditions in an aquatic ecosystem. These organisms make an ideal bioindicator since they are sessile in nature, have short generation times, and each species has its own set of environmental tolerances and preferences (Dunn *et al*. 2008). Moreover, these organisms are dominant species in the lotic water system and play a significant role in ecological balance between several types of macrophytes and their respective aquatic environment (Hassan *et al*. 2014; Fawzy 2016).

Aquatic macrophytes colonise the soft, sandy sediments of freshwater habitats and are key contributors to the primary productivity of the autotrophic community in the aquatic ecosystems. The relationships between host plants and attached microalgae in the natural environment are still incompletely understood (Toporowska *et al*. 2008). Most macrophyte substrata are highly dynamic in their physical characteristics and their chemical contribution to attached algal flora (Wetzel 1983). Free-floating macrophytes such as water hyacinth (*Eichhornia crassipes*) are able to take over light and consume nutrients from the water column, prohibiting phytoplankton from obtaining sufficient resources for photosynthesis (McVea & Boyd 1975). Thus, free-floating macrophytes can dominate phytoplankton and other submerged vegetation. Aquatic plants may also reveal allelopathic activity against epiphytic algae, and as a result the development of epiphyton depends on macrophyte host species (Toporowska *et al*. 2008). On the other hand, epiphyton can reduce growth and production of macrophytes due to faster uptake of nutrients by epiphytic microalgae than by plant. Macrophytes can cause increase in population of phytoplankton in an aquatic ecosystem by entrapping detritus material in their roots thereby increasing phytoplankton density beneath mats. Overall, free-floating macrophytes seems to limit the productivity of phytoplankton and submersed vegetation under mats, with the exception of certain colonial algal types that may initially be captured within the roots of water hyacinth (Villamagna 2009).

Studies concerning taxonomy and species composition of epiphytic algae on different macrophytes (such as *E. crassipes*) are still limited in the Philippines. The aim of this paper was to study the species composition of epiphytic algae (non-diatom) on the submerged parts of a macrophyte (*Eichhornia crassipes*) inhabiting the littoral zone of Laguna de Bay.

## MATERIALS AND METHOD

A single preliminary collection of epiphytic algae from water hyacinth (*E. crassipes*) was made on 20 May 2017. The submerged leaves and roots of *E. crassipes* were placed in polyethylene bags and kept wet for laboratory examination. Separation of epiphytic algae population from their host was carried out by scraping and manual shaking for 30 minutes (Zimba & Hopson 1997). Photomicrographs of the algal isolates appearing in the culture medium (BG 11 medium with or without combined nitrogen) were taken using an AO Series 10 Microstar Microscope Binocular Model 10B (USA) and with an Olympus CX31 binocular research microscope (USA) equipped with Infinity X digital camera. The morphological characteristics such as attributes of the filaments, the size and shape of vegetative cells as well as specialized cells (heterocytes and akinetes), length and width of intercalary cells, absence or presence of constriction at the cross wall and at the sheath; colour and appearance of the sheath; nature of trichomes and filaments; absence or presence of heterocytes and akinete were taken into examination during the identification and classification of each algal taxa. The taxonomic system described by Tilden (1910), Desikachary (1959), Prescott (1962), Anagnostidis and Komárek (1990), Wolowski (2011) and Whitton (2011) were used. Morphotaxonomic identification was done to the lowest taxonomic level possible using all available information. In the current taxonomic study, the orthographs ‘heterocytes’ and ‘hormogonia’ instead of ‘heterocytes’ and ‘hormogones’ respectively were applied respectively, as proposed by the International Association for Cyanophyte Research (IAC) (Mollenhauer *et al*. 1994).

## RESULTS

A total of 21 epiphytic microalgae (belonging to Cyanophyceae, Chlorophyceae, Trebouxiophyceae, Klebsormidiophyceae and Euglenophyceae) were identified. The specimen were described and photographed for the first time in the studied area in order to fill the gap of information of epiphytic algae associated to water hyacinth found in Laguna de Bay (Philippines). Taxonomy based on morphological characterisation of each of the isolates is presented together with a short description of the place of collection and habitat of their occurrence. Current names were used based on Guiry and Guiry (2017).

### Taxonomic Enumeration

#### Cyanobacteria

Class: Cyanophyceae

Order: Oscillatoriales

Family: Oscillatoriaceae

##### Genus: *Oscillatoria* Vaucher ex Gomont

###### 1. *Oscillatoria limosa* C. Agardh *ex* Gomont (Pl. I, Fig. 1)

Desikachary (1959, p. 206, Pl. 42, Fig.11); Prescott (1962, p. 489, Pl. 109, Fig. 17); Pantastico (1977, p. 46, Pl. IV, Fig. 1); Whitton (2011, p. 99, Pl. 20G,H); Sethi *et al*. (2012, p. 3, Pl. 2, Fig. 3), Kesarwani *et al*. (2015, p. 26, Pl. 2, Fig. 19; Pl. 3, Fig. 38; Pl. 4, Fig. 47).

Trichomes scattered, straight, slightly constricted to crosswalls; apical cells rounded or flattened, not attenuated and not capitated without calyptra; heterocytes and akinetes are absent; cells blue-green in colour, 8–12 μm long and 2–4 μm wide, protoplasm finely granular; crosswalls often granulated.

Found occurring as a greenish crust on submerged roots associated with other filamentous cyanobacteria.

Specimen: LUZON, Laguna, Los Baños (Mayondon, Station 1), E.DLR. Arguelles *s.n*. Photograph prepared from the mounted specimen.

###### 2. *Oscillatoria tenuis* C. Agardh *ex* Gomont (Pl. I, Fig. 2)

Desikachary (1959, p. 222, Pl. 42, Fig. 15); Prescott (1962, p. 491, Pl. 110, Fig. 8,9,14); Pantastico (1977, p. 54, Pl. IV, Fig. 11); Whitton (2011, p. 101, Pl. 20K); Kesarwani *et al*. (2015, p. 25, Pl. 1, Fig. 9; Pl. 3, Fig. 40).

Trichomes scattered, straight or slightly bend in the apical few cells, 5–6 μm broad; slightly constricted to crosswalls; apical cells rounded or flattened, not attenuated towards apex and without calyptra; heterocytes and akinetes are absent; cells blue-green in colour, 1–3 μm long and 4–6 μm wide, crosswalls narrowed, protoplasm finely granular; crosswalls often granulated; end cell more or less hemispherical.

Found occurring as a slimy, blue-green film attached on the roots of water hyacinth associated with other filamentous cyanobacteria.

Specimen: LUZON, Laguna, Los Baños (Bayan, Station 2), E.DLR. Arguelles *s.n*. Photograph prepared from the mounted specimen.

##### Genus: *Phormidium* Kützing ex Gomont

###### 1. *Phormidium granulatum* (Gardner) Anagnostidis (Pl. I, Fig. 3)

Basionym: *Oscillatoria granulata* Gardner

Prescott (1962, p. 489, Pl. 109, Fig. 12,13); Pantastico (1977, p. 41, Pl. IV, Fig. 9); Whitton (2011, p. 99).

Trichomes cylindrical, more or less straight; not or very slightly constricted at the crosswall; apex not attenuated, motile; apical cells are usually rounded, without or with slightly thickened cell walls, calyptra absent; heterocytes and akinetes are absent; cells dark green in colour, isodiametric or sometimes shorter than wide, 2.0–4.0 (4.5) μm long, cells have distinct granules at the crosswalls.

Found occurring as a blackish green crust associated with other unicellular green algae.

Specimen: LUZON, Laguna, Los Baños (Mayondon, Station 1), E.DLR. Arguelles *s.n*. Photograph prepared from the mounted specimen.

Order: Synechococcales

Family: Leptolyngbyaceae

##### Genus: *Leptolyngbya* K. Anagnostidis & J. Komárek

###### 1. *Leptolyngbya lagerheimii* (Gomont ex Gomont) Anagnostidis & Komárek (Pl. I, Fig. 4)

Basionym: *Lyngbya lagerheimii* Gomont ex Gomont

Desikachary (1959, p. 290, Pl. 48, Fig. 6; Pl. 53, Fig. 2); Prescott (1962, p. 501, Pl. 112, Fig. 5,6); Whitton (2011, p. 91, Pl. 11D, M).

Trichomes clumped and slightly curled up. Filaments are blue green in colour, 2 μm long and 2–3 μm wide, protoplasm not granular, septa not granulated, apical cells rounded without calyptra; end cells rounded; sheaths 2 μm wide, colourless and strong.

Found occurring as a bluish green crust attached to submerged roots associated with other filamentous cyanobacteria.

Specimen: LUZON, Laguna, Los Baños (Mayondon, Station 1), E.DLR. Arguelles *s.n*. Photograph prepared from the mounted specimen.

Family: Pseudanabaenaceae

Genus: *Pseudanabaena* Lauterborn

###### 1. *Pseudanabaena minima* (G.S. An) Anagnostidis (Pl. I, Fig. 5)

Basionym: *Achroonema minimum*

Park (2012, p. 49, Figs. 17D–G).

Trichomes occur as solitary or crowded in clusters, straight, highly constricted at thickened crosswalls, end cells are not attenuated, 1.5–2.0 μm wide. Cells up to 2 times longer than wide, 2.0–4.0 μm long, blue-green to pale blue-green in colour; cell content uniform without aerotopes. Apical cell widely rounded and without aerotopes.

Found occurring as a bluish green crust attached to submerged roots associated with other green algae.

A new record for the Philippines.

Specimen: LUZON, Laguna, Los Baños (Mayondon, Station 1), E.DLR. Arguelles *s.n*. Photograph prepared from the mounted specimen.

Family: Synechococcaceae

##### Genus: Synechococcus Nägeli

###### 1. *Synechococcus nidulans* (Pringsheim) Komárek (Pl. I, Fig. 6)

Basionym: *Lauterbonia nidulans* Pringsheim

McGregor *et al*. (2007, p. 309, Fig. 15).

Cells solitary but sometimes in group not forming mucilaginous colonies; cells sometimes occur in short series (pseudofilamentous formation) with 2–10 cells. Mucilage absent or sometimes present very fine, colourless, diffluent, around single cells. Cells cylindrical or long oval, sometimes several times longer than wide, straight, 3 up to more than 8 μm long and 0.5–2 μm wide, with parietal chloroplasts.

Found occurring as a bluish green crust submerged roots associated with other filamentous cyanobacteria.

A new record for the Philippines.

Specimen: LUZON, Laguna, Los Baños (Bayan, Station 2), E.DLR. Arguelles *s.n*. Photograph prepared from the mounted specimen.

Order: Nostocales

Family: Hapalosiphonaceae

##### Genus: *Hapalosiphon* Nägeli ex É. Bornet & C. Flahault

###### 1. *Hapalosiphon welwitschii* West & G.S.West (Pl. I, Fig. 7)

Desikachary (1959, p. 588, Pl. 137, Fig. 5); Pantastico (1977, p. 72, Pl. VI, Fig. 5); Saha *et al*. (2007, p. 47: 219, Fig. 29); Arulmurugan *et al*. (2011 p. 28, Pl. 3, Fig. 12); Arguelles (2016, p. 30, Pl. I, Fig. 5).

Filaments are uniserial having trichomes displaying true branches; 5–6 μm broad, straight, slightly constricted at the crosswalls; sheath is colourless and slightly visible; apex is slightly attenuated and capitated; lateral branches are short, as broad as the main filament narrower; cells blue-green in colour and are cylindrical, 3 μm long and 4 μm wide, protoplasm not granular, septa not granulated, end cells rounded; heterocytes rare, intercalary, rounded or cylindrical.

Found occurring as a dark greenish crust on leaves slightly submerged in water associated with other cyanobacteria.

Specimen: LUZON, Laguna, Los Baños (Bayan, Station 2), E.DLR. Arguelles *s.n*. Photograph prepared from the mounted specimen.

#### Chlorophyta

Class: Chlorophyceae

Order: Chlamydomonodales

Family: Chlorococcaceae

##### Genus: *Chlorococcum* Meneghini

###### 1. *Chlorococcum infusionum* (Schrank) Meneghini (Pl. II, Fig. 1)

Synonym: *Chlorococcum humicola* (Nägeli) Rabenhorst 1868

Basionym: *Cystococcus humicola* Nägeli

Prescott (1962, p. 280, Pl. 45, Fig. 1); Pantastico (1977, p. 76, Pl. VII, Fig. 1); Zafaralla (1998, p. 33, Pl, 8e, f); Samad & Adhikary (2008, p. 91, Pl. 1, Fig. 1); John (2011, p. 414, Pl. 103L); Arguelles (2016, p. 17: 32, Pl. I, Fig. 7).

Spherical cells, usually solitary but sometimes several cells are congested together to form a cluster of cells, greenish in colour; parietal chloroplast (with a single pyrenoid) nearly wrapping the entire cells; cells 9–12 μm in diameter.

Found occurring as a greenish crust on submerged roots associated with other cyanobacteria.

Specimen: LUZON, Laguna, Los Baños (Mayondon, Station 1), E.DLR. Arguelles s.n. Photograph prepared from the mounted specimen.

Order: Sphaeropleales

Family: Scenedesmaceae

##### Genus: *Tetradesmus* G.M.Smith

###### 1. *Tetradesmus obliquus* (Turpin) M.J.Wynne (Pl. II, Fig. 2)

Basionym: *Achnanthes obliqua* Turpin

*Scenedesmus obliquus* (Turpin) Kützing

Prescott (1962, p. 279, Pl. 63, Fig. 17); Jena & Adhikary (2007, p. 181, Pl. 3, Figs. 25–26); Kim (2015, p. 34, Figs. 17, 18A–O); Bose *et al*. (2016, p. 85, Figs. 3 f, g).

Colonies with 2–4 cells attached side by side, arranged linearly or alternating cells (in 1 or 2 rows) forming a coenobia; cells uninucleated, spindle-shaped, 2–4 μm long and 4–6 μm wide, with a parietal chloroplast and a single pyrenoid; inner cells are straight while the terminal cells are arcuate; cell walls are smooth without spine or granulates.

Found occurring as a dark greenish crust on leaves slightly submerged in water associated with other cyanobacteria.

Specimen: LUZON, Laguna, Los Baños (Mayondon, Station 1), E.DLR. Arguelles *s.n*. Photograph prepared from the mounted specimen.

##### Genus: *Acutodesmus* (Hegewald) Tsarenko

###### 1. *Acutodesmus dimorphus* (Turpin) Tsarenko (Pl. II, Fig. 3)

Basionym: *Achnanthes dimorpha* Turpin

Synonyms: *Scenedesmus acutus* Meyen, *Scenedesmus antennatus* Brébisson, *Scenedesmus costulatus* Chodat

John (2011, p. 421, Pl. 104B,112M).

Colonies with 2–4 cells attached side by side, arranged linearly or alternating cells (in 1 or 2 rows) forming a coenobia; cells uninucleated, spindle-shaped, 2–4 μm long and 4–6 μm wide, with a parietal chloroplast and a single pyrenoid; inner cells are straight while the terminal cells are arcuate; cell walls are smooth without spine or granulates.

Found occurring as a greenish froth on leaves submerged in water.

Specimen: LUZON, Laguna, Los Baños (Mayondon, Station 1), E.DLR. Arguelles *s.n*. Photograph prepared from the mounted specimen.

Family: Hydrodictyaceae

##### Genus: *Pseudopediastrum* E. Hegewald

###### 1. *Pseudopediastrum boryanum* (Turpin) E. Hegewald (Pl. II, Fig. 4)

Synonym: *Pediastrum boryanum* (Turpin) Meneghini

Basionym: *Helierella boryana* Turpin

Prescott (1962, p. 222, Pl. 47, Fig. 9); Philipose (1967, p. 118, Fig. 40); Pantastico (1977, p. 87, Pl. VII, Fig. 10); Perez *et al*. (2002, p. 103, 61(2): Figs. 2.22, 2.68); John (2011, p. 463, Pl. 119I); Al-Hassany & Hassan (2015, p. 99, Pl. 2, Fig. 11).

Coenobium circular to oval, usually 16–32 celled, up to 188 celled; cells arranged in concentric rings without intercellular space; inner cells polygonal with straight sides, outer face of peripheral cells slightly to deeply emarginate and with two short spines; cell wall usually granulated or sometimes smooth; cells up to 38 μm in diameter, processes 15–30 μm long; colony 49 μm in diameter.

Found occurring as a greenish froth on leaves submerged in water associated with other cyanobacteria.

Specimen: LUZON, Laguna, Los Baños (Bayan, Station 2), E.DLR. Arguelles *s.n*. Photograph prepared from the mounted specimen.

##### Genus: *Pediastrum* Meyen

###### 1. *Pediastrum duplex* Meyen (Pl. II, Fig. 5)

Prescott (1962, p. 232, Pl. 48, Fig. 4); Tiffany and Britton (1971, p. 952, 112, Pl. 30, Fig. 300); Philipose (1967, p. 121, Fig. 43b); Perez (2002, p.103, Fig. 2.24); John (2011, p. 463, Pl. 119C); Rai & Misra (2012, p. 170, Figs. 8–9).

Colony of 16–32 celled, sometimes 4, 8, 64 or 128 celled with small lens shaped perforations between cells; inner cells quadrate to angular in shape and not in contact at the central part of the side walls, inner side of marginal cells concave, outer side produced into two short blunt-tipped processes; colonies 45–93 μm in diameter; marginal cells 12 μm long, 8–12 μm broad; inner cells 10 long, 8–9 μm broad.

Found occurring as light greenish froth on roots submerged in water.

Specimen: LUZON, Laguna, Los Baños (Mayondon, Station 1), E.DLR. Arguelles *s.n*. Photograph prepared from the mounted specimen.

Class: Trebouxiophyceae

Order: Chlorellales

Family: Chlorellaceae

##### Genus: *Chlorella* Beyerinck [Beijerinck]

###### 1. *Chlorella vulgaris* Beyerinck [Beijerinck] (Pl. II, Fig. 6)

Basionym: Chlorella pyrenoidosa var. duplex (Kützing)

Prescott (1962, p. 237, Pl. 53, Fig. 13); Ortega-Calvo *et al*. (1993, p. 246, Pl. 2, Figs. 16 and 17); Sethi *et al*. (2012, p. 3, Pl. 3, Fig. 27); Satpati *et al*. (2013, p. 30, Pl. 1, Fig. 1 and Pl. 5, Fig. 2).

Spherical or almost spherical cells; thin cell wall; chloroplast is single, parietal and cup-shaped with only one spherical pyrenoid occupying a basal zone of the cell; young cells either ellipsoidal or spherical, 1.0 x 2.0 μm or 2.5 μm in diameter; cell reproduction is by formation of 2 or 4 autospores of the same size, set free by the rupture of mother cell wall.

Found occurring as a greenish crust on submerged roots associated with other filamentous cyanobacteria.

Specimen: LUZON, Laguna, Los Baños (Mayondon, Station 1), E.DLR. Arguelles *s.n*. Photograph prepared from the mounted specimen.

##### Genus: *Micractinium* Fresenius

###### 1. *Micractinium pusillum* Fresenius (Pl. II, Fig. 7)

Synonym: *Golenkinia botryoides* Schmidle

Pantastico (1977, p. 77, Pl. VII, Fig. 2); Philipose (1967, p. 104, Fig. 19); Prescott (1962, p. 287, Pl. 66, Fig. 8); Tiffany & Britton (1971, p. 106, Pl. 33, Fig. 329); John (2011, p. 488, Pl. 121L); Perez *et al*. (2002, p. 103, Figs. 2.23, 2.57).

Colony of 4–16 spherical cells arranged in a pyramid or in a square, groups of 4 in association with other similar groups; free walls beset with 2–3 finely tapering setae; chloroplasts a parietal cup with one pyrenoid; cells 4.2–6.8 μm in diameter without setae; setae 12 μm long.

Found occurring as a greenish crust on roots submerged in water associated with other green alga and cyanobacteria.

Specimen: LUZON, Laguna, Los Baños (Bayan, Station 2), E.DLR. Arguelles *s.n*. Photograph prepared from the mounted specimen.

#### Charophyta

Class: Klebsormidiophyceae

Order: Klebsormidiales

Family: Klebsormidiaceae

##### Genus: *Klebsormidium* P.C.Silva, Mattox & W.H. Blackwell

###### 1. *Klebsormidium flaccidum* (Kützing) P. C. Silva, K. R. Mattox & W. H. Blackwell (Pl. III, Fig. 1)

Basionym: *Ulothrix flaccida* Kützing

Pantastico (1977, p. 143, Pl. XI, Fig. 1); Ortega-Calvo *et al*. (1993, p. 246, Pl. 3, Fig. 32); Flechtner *et al*. (2008, p. 410, Fig. 5); John (2011, p. 556, Pl. 138J); Mikhailyuk *et al*. (2015, p. 755, Figs. 2a–f).

Filaments are normally long with tendency to separate and break apart into small fragments, slightly constricted; cells are often cylindrical with rounded ends, 13 μm long and 6 μm wide; cell wall moderately thickened; chloroplasts are parietal and band-shaped (with one pyrenoid) which covers 1/2–2/3 of the cell inner surface and periphery. In liquid media, this organism are capable of forming a surface hydrorepellent layer and submerged tufts; on agar forming irregular and undulating colonies.

Found occurring as a greenish mat on submerged leaves associated with other filamentous cyanobacteria and green microalgae.

Specimen: LUZON, Laguna, Los Baños (Bayan, Station 2), E.DLR. Arguelles *s.n*. Photograph prepared from the mounted specimen.

#### Euglenophyta

Class: Euglenophyceae

Order: Euglenales

Family: Phacaceae

##### Genus: *Phacus* Dujardin

###### 1. *Ph*a*cus longicauda* (Ehrenberg) Dujardin (Pl. III, Fig. 2)

Synonym: *Phacus longicauda* var. *major* Svirenko

Basionym: *Euglena longicauda* Ehrenberg

Prescott (1962, p. 400, Pl. 87, Fig. 1); Wolowski *et al*. (2013, p. 676, Fig. 60); Pantastico (1977, p. 176, Pl. XV, Fig. 4); Wolowski (2011, p. 210, Pl. 52L); Arguelles *et al*. (2014, p. 17, Pl. II, Fig. 1).

Cells are widely obovoid and flattened (91.0 μm × 47.5 μm) in outline; anterior pole is broadly rounded, sometimes bilobed; posterior end narrowed and gradually tapering into a long, straight cauda; several small discoid chloroplasts; one large paramylon body in the centre, accompanied by 1–3 smaller ones; pellicle is spirally striated.

Found occurring as a greenish crust on submerged leaves associated with other planktonic algae.

Specimen: LUZON, Laguna, Los Baños (Bayan, Station 2), E.DLR. Arguelles *s.n*. Photograph prepared from the mounted specimen.

###### 1. *Phacus pleuronectes* (Ehrenberg) Dujardin (Pl. III, Fig. 3)

Synonym: *Cerasteria pleuronectes* Müller

Wolowski *et al*. (2013, p. 674, Figs. 53, 94, 110); Wolowski (2011, p. 212, Pl. 53E); Arguelles *et al*. (2014, p. 19, Pl. II, Fig. 5).

Cells suborbicular (36 μm × 67.5 μm) and are slightly symmetric; anterior end narrowly rounded; apical furrow up to half cell length; posterior end with short curved tail-piece turning obliquely to one side; pellicle is longitudinally striated; several parietal chloroplasts are present in the cytoplasm; eyespot conspicuous; flagellum equal to or longer than cell length.

Found occurring as a light greenish crust on submerged roots associated with other filamentous green algae and cyanobacteria.

Specimen: LUZON, Laguna, Los Baños (Bayan, Station 2), E.DLR. Arguelles *s.n*. Photograph prepared from the mounted specimen.

##### Genus: *Lepocinclis* Perty

###### 1. *Lepocinclis fusiformis* (Carter) Lemmermann (Pl. III, Fig. 4)

Synonym: *Lepocinclis sphagnophila* Lemmermann

Basionym: *Euglena fusiformis* Carter

Wolowski (2011, p. 198, Pl. 50I).

Cells lemon-shaped, 29.0–29.5 μm long, 23.0–26.6 μm wide; anterior pole conically narrowing with small concavity; posterior pole narrowing with short conical tail-piece; pellicle colourless with left-handed striae; numerous discoid chloroplasts; two thick annular paramylon bodies, lateral and opposite.

Found occurring as a dark greenish froth on submerged stem and leaves associated with other filamentous cyanobacteria.

Specimen: LUZON, Laguna, Los Baños (Mayondon, Station 1), E.DLR. Arguelles *s.n*. Photograph prepared from the mounted specimen.

Family: Euglenaceae

##### Genus: *Euglena* Ehrenberg

###### 1. *Euglena agilis* Carter (Pl. III, Fig. 5)

Synonym: *Euglena pisciformis* Klebs

Boonmee *et al*. (2011, p. 119, Pl. II, Fig. 2); Wolowski (2011, p. 187, Pl. 35G, H); Wolowski *et al*. (2013, p. 666, Figs. 18, 80).

Cells are highly metabolic, cylindrical (24 μm × 11 μm); anterior end is bluntly rounded when fully extended; posterior end coming to a short rather blunt point; with two chloroplasts (elongated widened plates containing pyrenoids which are sheathed with paramylum cups) per cell; pellicle is faintly striated; flagellum approximates the body length.

Found occurring as a greenish crust on submerged roots associated with other planktonic algae.

Specimen: LUZON, Laguna, Los Baños (Mayondon, Station 1), E.DLR. Arguelles *s.n*. Photograph prepared from the mounted specimen.

##### Genus: *Trachelomonas* Ehrenberg

###### 1. *Trachelomonas volvocina* Ehrenberg (Pl. III, Fig. 6)

Basionym: *Microglena longicauda* Ehrenberg

Smith (1950, p. 356, Figs. 259, 1950); Pringsheim (1953, pp. 244–245, Fig. 9); Prescott (1962, p. 419, Pl. 83, Figs. 1, 7, 8); Whitford & Schumacher (1969, Pl. 44, Fig. 3); Tiffany and Britton (1971, p. 326, Pl. 88, Fig. 1026); Pantastico (1977, p. 185, Pl. 16, Fig. 7); Boonmee *et al*. (2011, pp. 99–141, Pl. I, Fig. 5); Wolowski (2011, p. 226, Pl. 57A); Arguelles *et al*. (2014, p. 26, Pl. III, Fig. 8).

Lorica are spherical and smooth (23.5–24.0 μm in diameter), flagellum at the anterior part without a collar or slightly thickened around margin, smooth wall, reddish- brown; flagellum 2–3 times lorica length; chloroplasts 2 per cell.

Found occurring as a greenish film on submerged leaves associated with other filamentous algae.

Specimen: LUZON, Laguna, Los Baños (Bayan, Station 2), E.DLR. Arguelles *s.n*. Photograph prepared from the mounted specimen.

##### Genus: *Strombomonas* Deflandre

###### 1. *Strombomonas acuminata* (Schmarda) Deflandre (Pl. III, Fig. 7)

Basionym: *Lagenella acuminata* Schmarda

Wolowski and Walne (2007, p. 413, Figs. 3, 75); Wolowski (2011, p. 213, Pl. 54A–H); Arguelles *et al*. (2014, p. 29, Pl. I, Fig. 10).

Lorica smooth, slightly trapezoid or triangular (31.0 μm × 23.0 μm); anterior pole is distinctly narrowed terminating in a collar that is short and obliquely truncate; posterior pole with prominent straight curved extension; several discoid chloroplasts are scattered in the cell; eyespot is present and is moderately large; flagellum is 2–3 times cell length.

Found occurring as a dark greenish film on submerged leaves and stems associated with other planktonic algae.

Specimen: LUZON, Laguna, Los Baños (Bayan, Station 2), E.DLR. Arguelles *s.n*. Photograph prepared from the mounted specimen.

## DISCUSSION

Microscopic examination of submerged roots and leaf fragments showed that fungi, diatoms, and detritus formed a principal part of the epiphyton of *E. crassipes* roots. Fungal hyphae habitually proliferated over the whole root and leaf surface allowing live microalgae to form only a minor portion of the epiphyton. This finding is similar to the observation of Rañola *et al*. (1990) on their study on the algal epiphytes of submerged macrophytes in Laguna de Bay. Based on the result of the sampling made, a diverse number of species of algae constitute the epiphyton of the submerged leaf and roots of *E. crassipes*. A total of 21 taxa belonging to 8 orders, 13 families, 19 genera and 21 species based on recent combined taxonomical approach were described. Of these taxa, the occurrence of two rare cyanobacteria, *Pseudanabaena minima* (G. S. An) Anagnostidis and *Synechococcus nidulans* (Pringsheim) Komárek are reported for the first time in the Philippines. Two species are also reported here for the first time in the Philippines based on current taxonomic nomenclature and these are *Pseudopediastrum boryanum* (Turpin) E. Hegewald and *Phormidium granulatum* (Gardner) Anagnostidis. These species were also found in other habitat from different parts of the country but were reported as *Pediastrum boryanum* (Turpin) Meneghini, *Oscillatoria granulata* Gardner, respectively.

Some of the microalgal genera reported in this study (eg. *Tetradesmus, Euglena, Oscillatoria, Phacus* and *Chlorella*) are usually associated with organically polluted enriched waters (Effiong & Inyang 2015). These epiphytons can be considered as good indicators of water quality and environmental changes due to their sensitivity to external sources of fertilisation. More work should be done to study in detail the effect of different environmental parameters on the spatial-temporal distribution of epiphytic algae on macrophytes. The ecological relationships between host aquatic macrophyte and attached algae in the natural environment can be further studied by doing comprehensive taxonomic studies of different epiphytic algae present in water hyacinth and other aquatic macrophytes found on different sampling areas in Laguna de Bay and do correlation studies on the different physical and chemical environmental factors (pH, dissolved oxygen, light intensity and the like) that affects the distribution pattern of epiphytic microalgae. The taxonomic record of epiphytic algae obtained from this study provides basal knowledge for the advancement of the taxonomy of epiphytic algae attached to a macrophyte (*E. crassipes*) in Laguna de Bay.

## CONCLUSION

The preliminary taxonomic survey presented in this paper provides a collection of algal epiphytes associated with *E. crassipes*, an abundant and common aquatic macrophyte found in the shallow waters of Laguna de Bay. The need to conserve algal genetic resources of less studied habitats such as aquatic macrophytes are very important since taxonomic survey and analyses are necessary to understand the role and interaction of these microalgae in several aquatic environments. Further studies are needed to establish diversity, taxonomy, distribution, and ecology of algal epiphytes in other aquatic macrophytes (eg. seagrass, seaweeds, freshwater macrophytes such as *Hydrilla, Ipomoea* and the like) found in marine and freshwater environments in the country.

## Figures and Tables

**Plate I:**
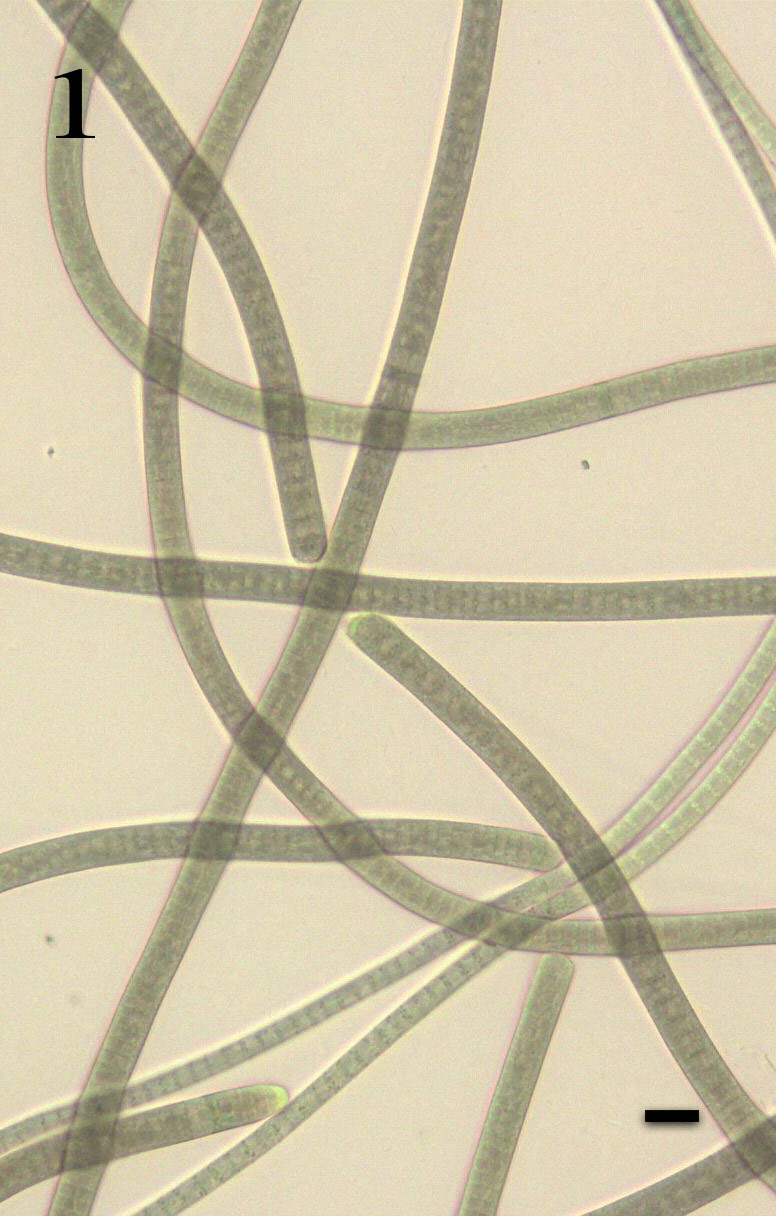

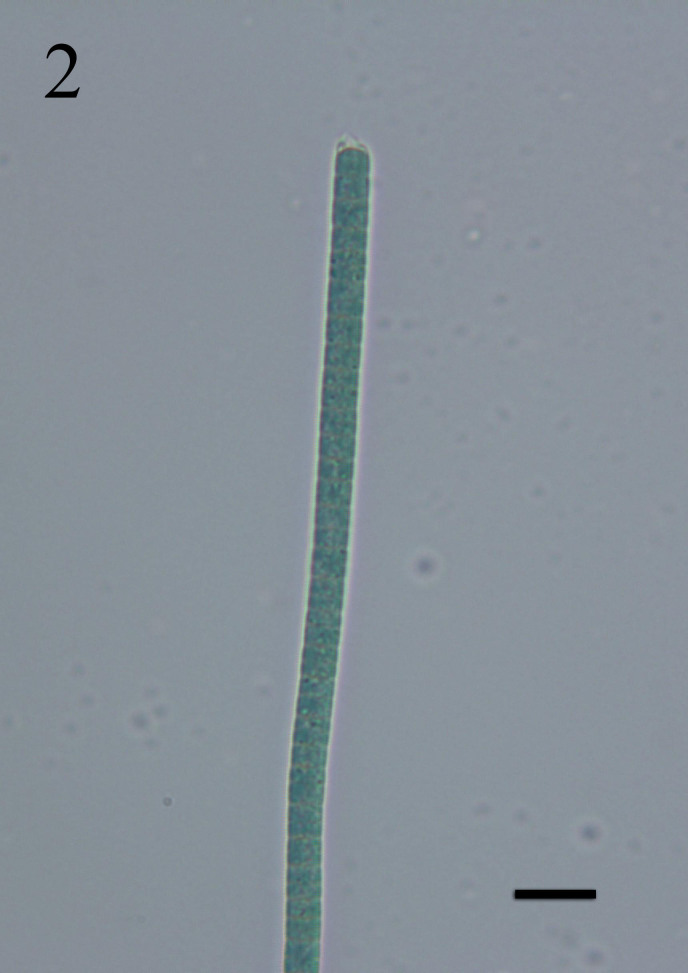

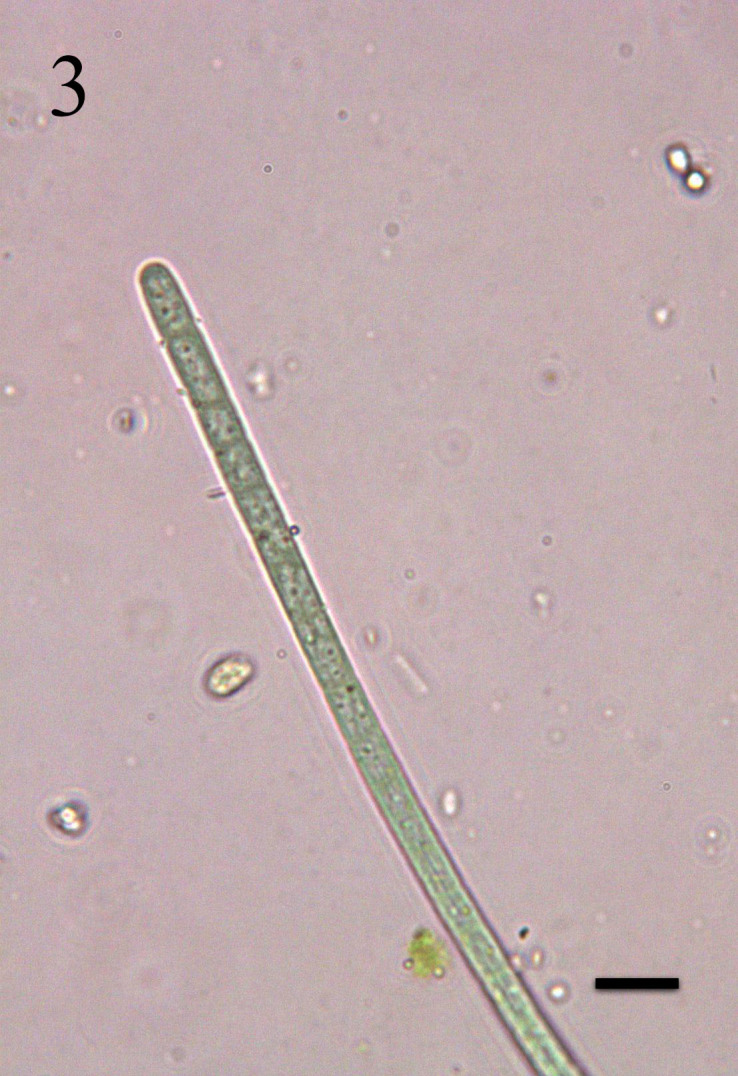

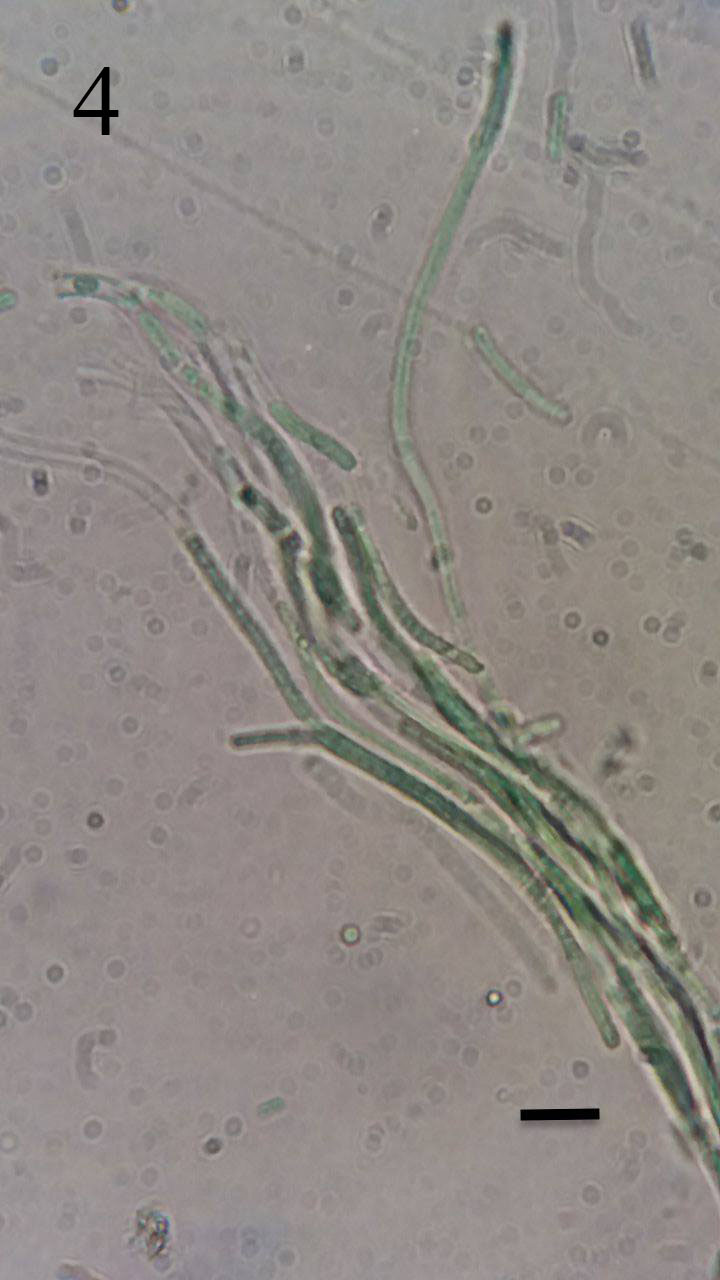

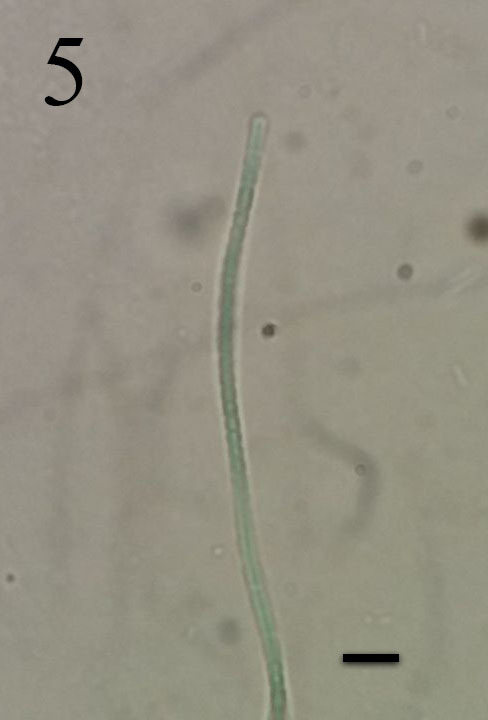

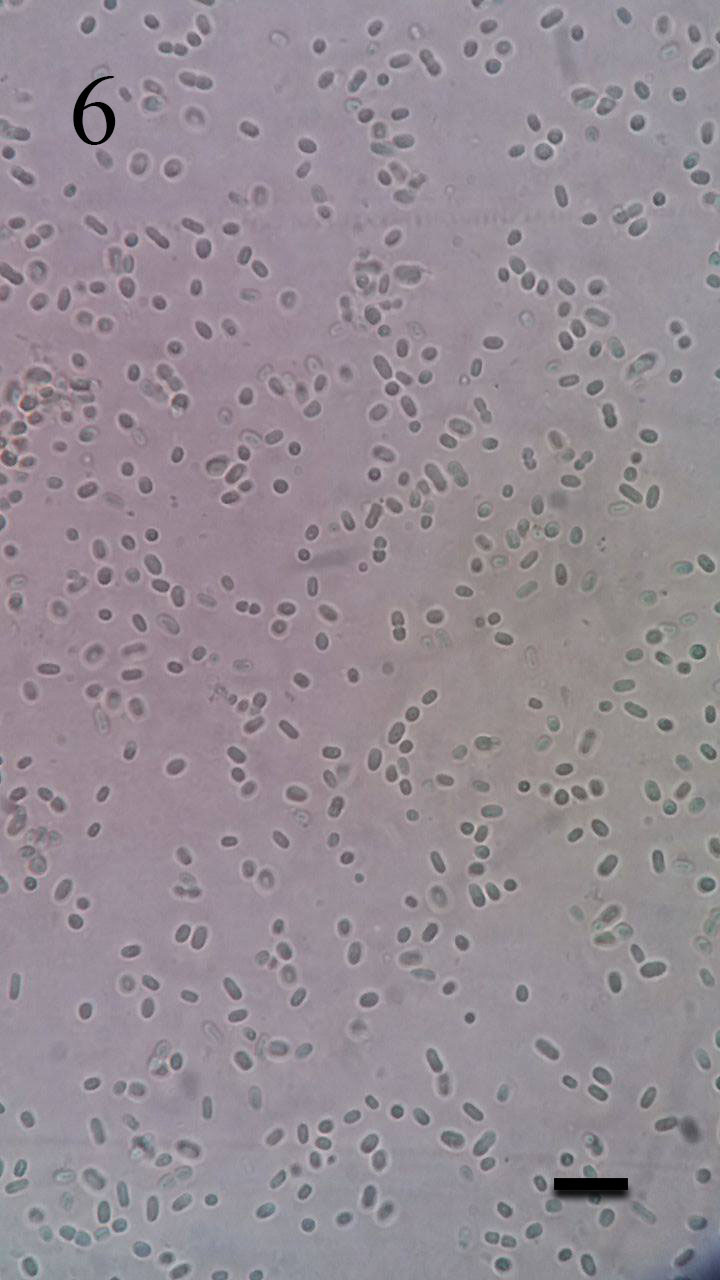

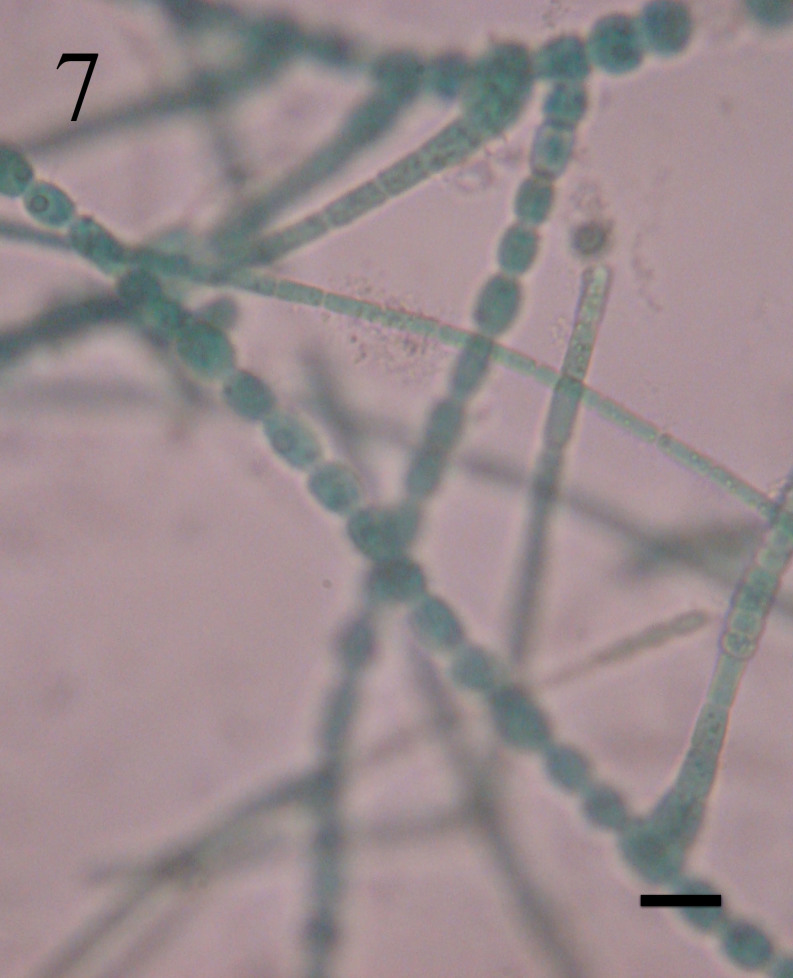
Fig. (1) *Oscillatoria limosa* C. Agardh *ex* Gomont; Fig. (2) *Oscillatoria tenuis* C. Agardh *ex* Gomont; Fig. (3) *Phormidium granulatum* (Gardner) Anagnostidis; Fig. (4) *Leptolyngbya lagerheimii* (Gomont ex Gomont) Anagnostidis; Fig. (5) *Pseudanabaena minima* (G.S. An) Anagnostidis & Komárek; Fig. (6) *Synechococcus nidulans* (Pringsheim) Komárek; Fig. (7) *Hapalosiphon welwitschii* West & G. S. West. All scale bars = 10 μm.

**Plate II:**
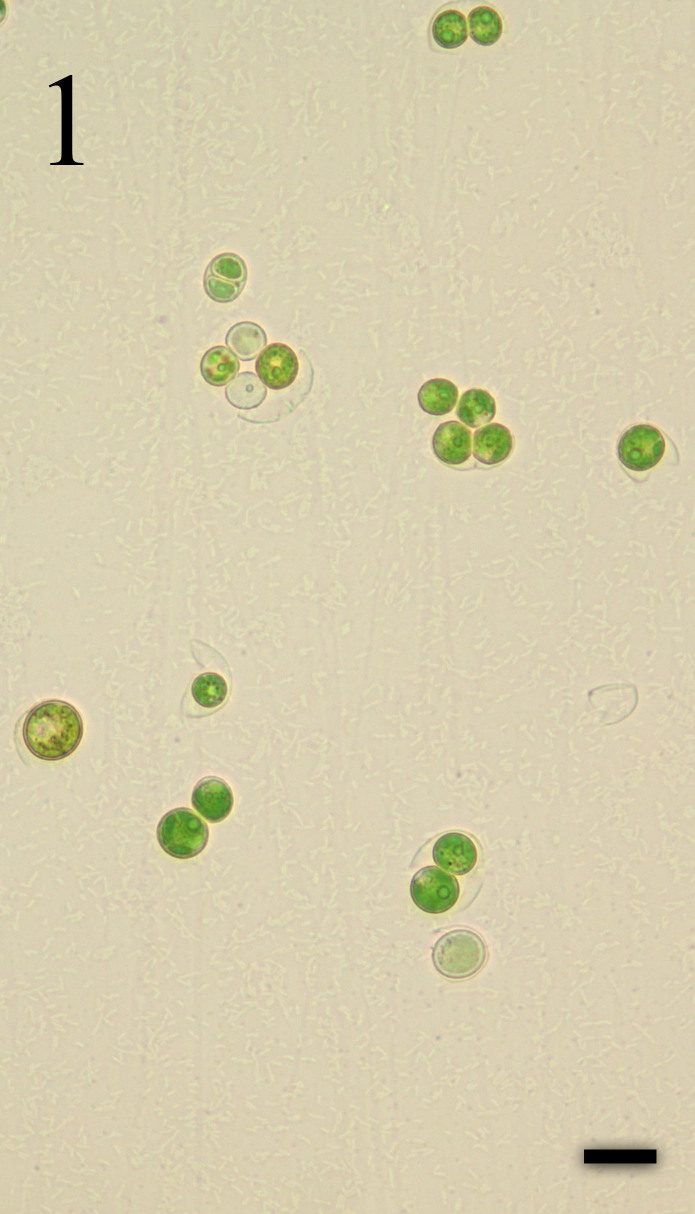

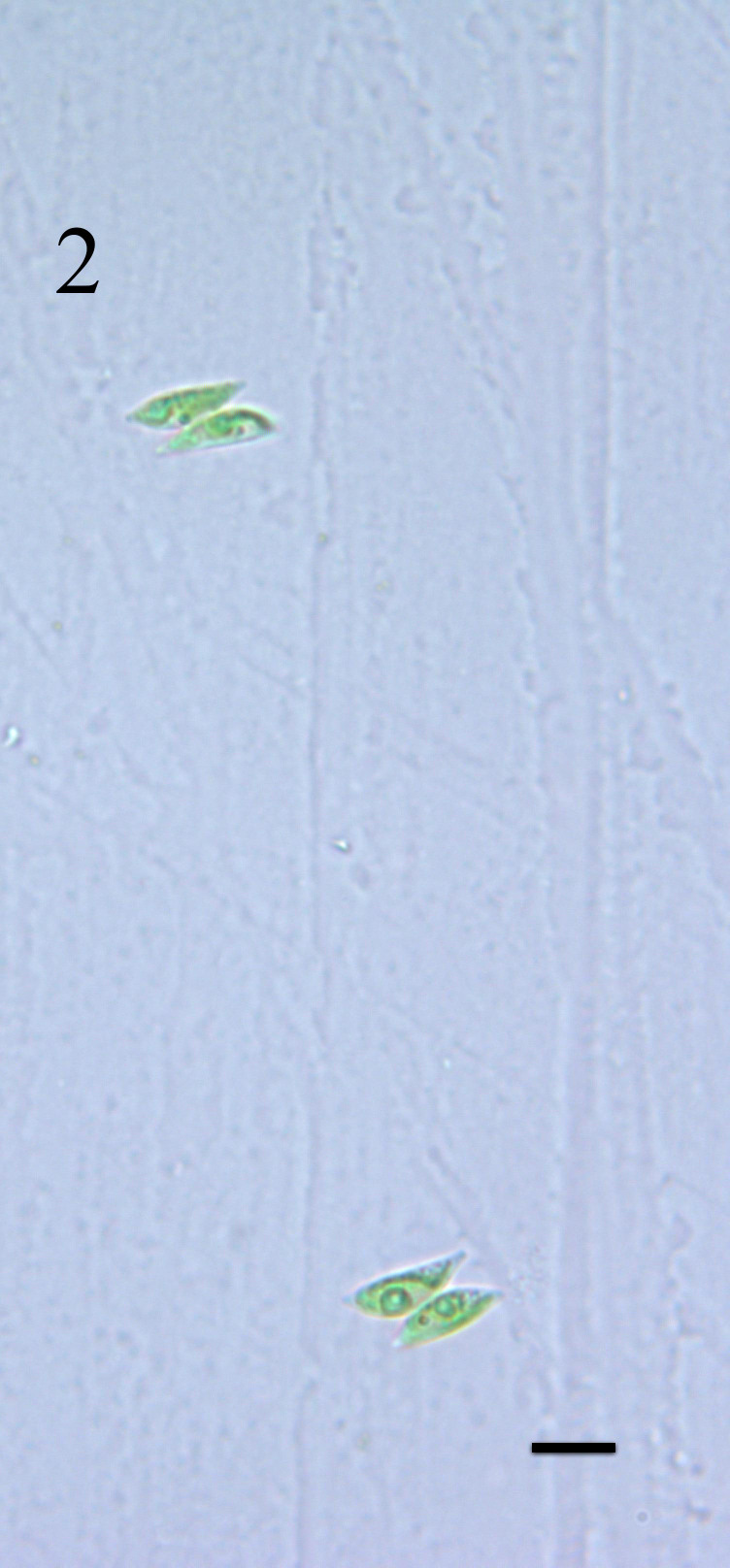

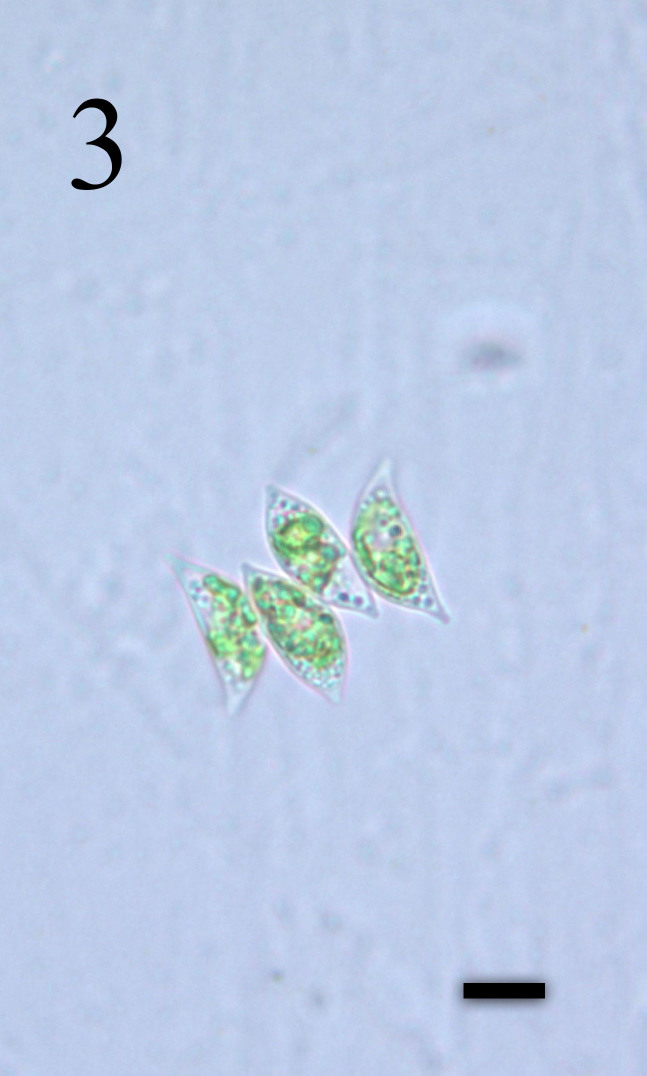

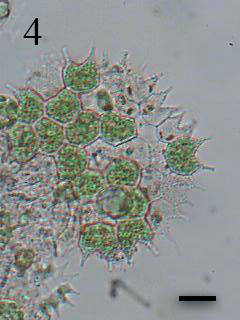

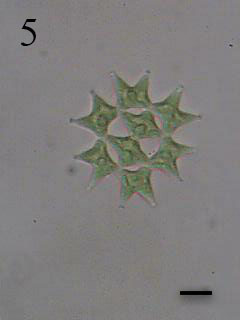

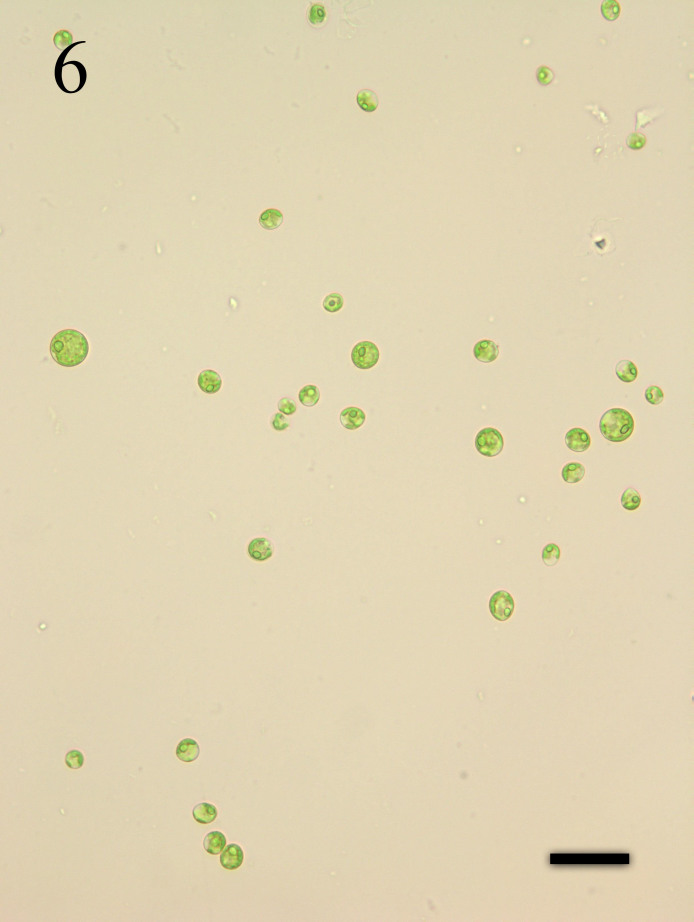

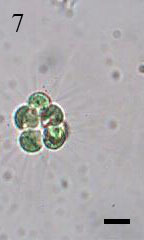
Fig. (1) *Chlorococcum infusionum* (Schrank) Meneghini; Fig. (2) *Tetradesmus obliquus* (Turpin) M.J.Wynne; Fig. (3) *Acutodesmus dimorphus* (Turpin) Tsarenko; Fig. (4) *Pseudopediastrum boryanum* (Turpin) E. Hegewald; Fig. (5) *Pediastrum duplex* Meyen; Fig. (6) *Chlorella vulgaris* Beyerinck [Beijerinck]; Fig. (7) *Micractinium pusillum* Fresenius. All scale bars = 10 μm.

**Plate III:**
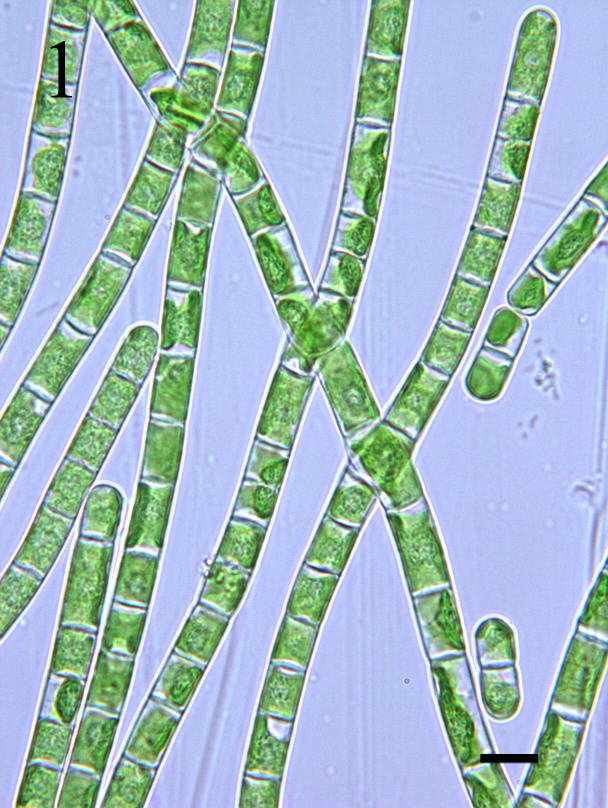

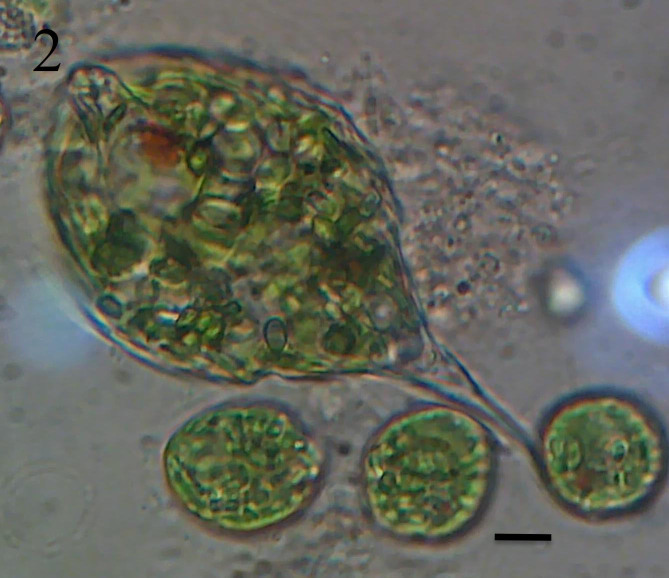

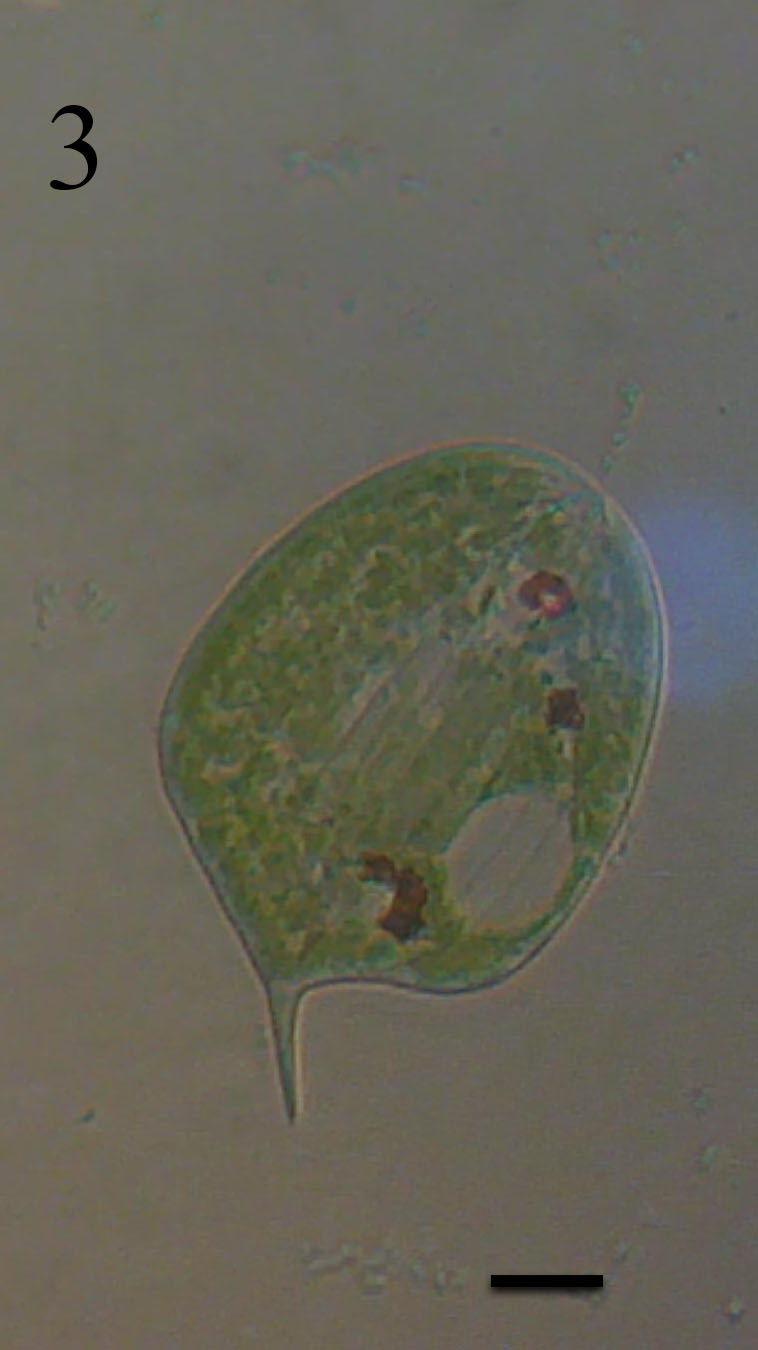

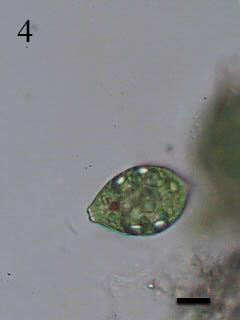

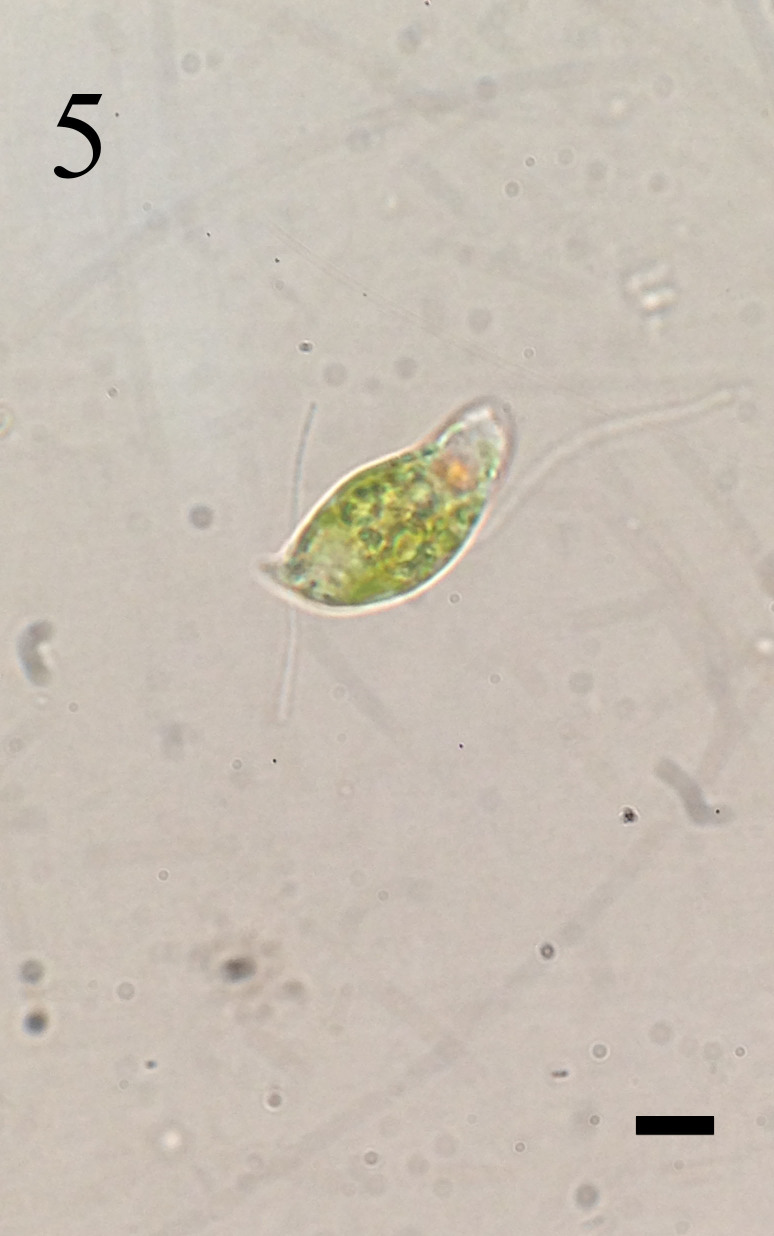

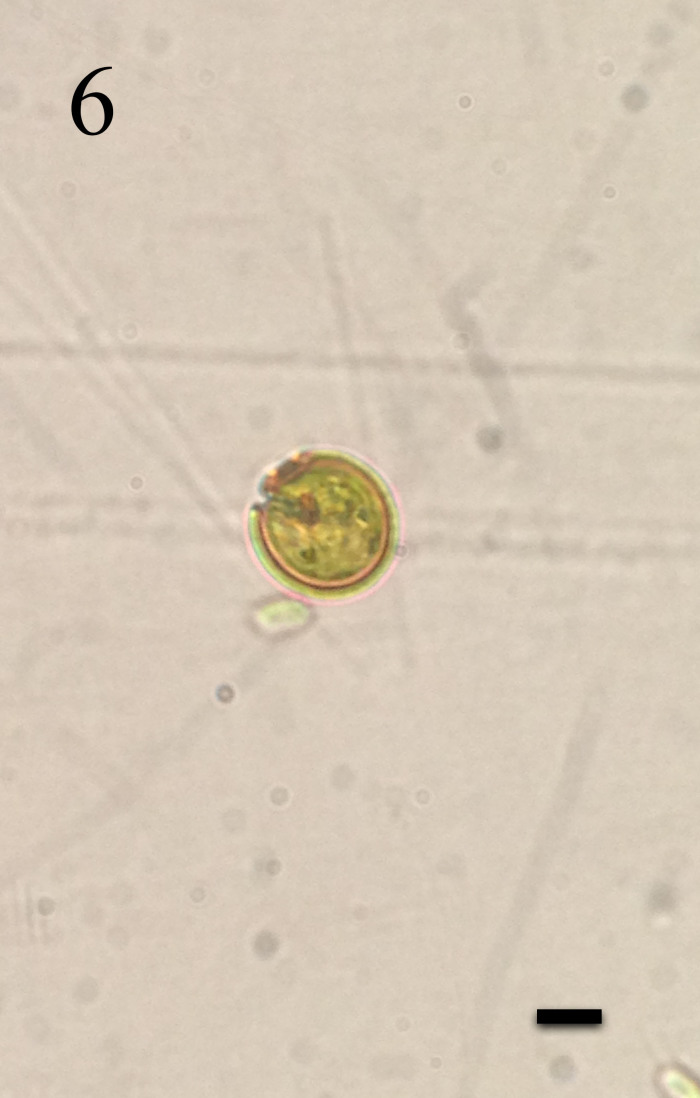

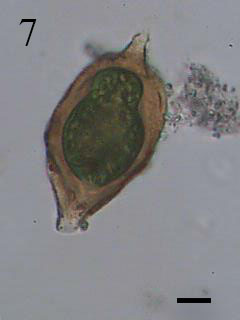
Fig. (1) *Klebsormidium flaccidum* (Kützing) P.C.Silva, K.R.Mattox & W.H.Blackwell; Fig. (2) *Phacus longicauda* (Ehrenberg) Dujardin; Fig. (3) *Phacus pleuronectes* (Ehrenberg) Dujardin; Fig. (4) *Lepocinclis fusiformis* (Carter) Lemmermann; Fig. (5) *Euglena agilis* Carter; Fig. (6) *Trachelomonas volvocina* Ehrenberg; Fig. (7) *Strombomonas acuminata* (Schmarda) Deflandre. All scale bars = 10 μm.
